# Complicated illegal induced abortions at a tertiary health institution in Nigeria

**DOI:** 10.12669/pjms.306.5506

**Published:** 2014

**Authors:** Maduabuchi Eugene Ikeanyi, Chukwunwendu Anthony Okonkwo

**Affiliations:** 1 Dr. Maduabuchi Eugene Ikeanyi, MBBS; FWACS, Department of Obstetrics and Gynaecology, Niger Delta University, Wilberforce Island, Yenagoa, Bayelsa State, Nigeria.; 2 Dr. Chukwunwendu Anthony Okonkwo, MBBS; FMCOG; FICS; D MAS, Department of Obstetrics and Gynaecology, University of Benin Teaching Hospital, Benin City Nigeria.

**Keywords:** Induced abortions, Complications

## Abstract

***Background and Objective:*** Globally it is estimated that 26-53 million induced abortions occur annually. An estimated 20 million of these are unsafe especially in countries with restrictive abortion laws. Approximately 48% of all abortions worldwide were unsafe and more than 97% of these are in developing countries. Our objective was to find out complications of illegal induced abortions in a tertiary care institution.

***Methods*** *: *All cases of complicated induced abortion, seen over a 5 year period were reviewed. Relevant data relating to the socio-demographic profile of the patients, clinical presentation, abortion service providers and facilities and mode of termination of pregnancy were extracted.

***Results:*** One hundred and nineteen patients, constituting 3.4% of gynaecological admissions were studied. The mean age of the patients was 23.5±6.6 years with over 80% single. The mean gestational age at abortion was 12.8± 4.1 weeks. Incomplete abortion and postabortal sepsis formed the major indication for admission. About a fifth of the cases had abdominal visceral involvement. Twenty (18%) had laparotomy and 10(9%) had renal dialysis. Over 75% of patients were discharged in stable state.

***Conclusion:*** This study highlights the pressing need for an organised program for reproductive health education especially for the adolescents and unmarried who were most affected by abortion complications. In addition training and continuing medical education for doctors favourably disposed to abortion services is highly indicated from this study.

## INTRODUCTION

Globally, it is estimated that 70,000 women, approximately 13% of annual maternal deaths result from complications of induced abortion with 99% of these in developing countries and 23,000 in African countries.^1^ In Nigeria, an estimated 20- 40% of maternal deaths result from abortion complications^2^^-^^4^ with a procedure-related death rate of 680 per 100,000 abortion.^5^

Induced abortion, an age-long method of fertility control is practiced in all continents and by all people.^6^ Induced abortion can be safe or unsafe. According to the World Health Organization unsafe abortion is the termination of an unintended pregnancy either by persons lacking the necessary skills or in an environment lacking the minimal medical standards or both.^7^

Reliable data on incidence of induced abortion and its complications is difficult to ascertain especially in areas with restrictive abortion laws.^8^ Globally it is estimated that 26-53 million induced abortions occur annually.^9^ An estimated 20 million of these are unsafe especially in countries with restrictive abortion laws. In 2003 alone, 48% of all abortions worldwide were unsafe and more than 97% of these were in developing countries.^10^ World abortion rate was calculated to be 35 per 1000 women aged 15-44, lowest 11/1000 in western Europe through 16/1000 in USA.^11^ to 33-37/1000 in Africa, Asia and Latin America with highest rate of 78-90 per 1000 in Cuba, Vietnam and E. Europe. It is estimated that each year Nigerian women obtain about 610,000 abortions, a rate of 25 abortions per 1000 women aged 15-44.^12^

Unintended pregnancy and birth could have negative consequences for women, children, families and societies. A significant proportion of women therefore opt for induced abortion to avoid unwanted births. In African setting unwanted pregnancy is a social stigma especially with the unmarried who goes to any length to have induced abortion. Lack of resources and support to take care of the babies, the fear of school drop-out and fear of social reprisal of premarital childbirths also compel most of the adolescent victims to go for induced abortion.^13^ Unmarried adolescents and women especially those with low education constitute the highest risk for unwanted pregnancies and induced abortion.^11^

Induced abortion is high and unsafe where abortion is highly restricted as in the poor developing countries of Africa, South and Central Asia and South America/Caribbean and quite low and safe where the procedure is legal and widely available as desired in the rich industrialized countries of the world^6^ With the exception of Zambia, Ghana and South African most of the sub-Saharan African countries have restrictive abortion laws. Nigeria has a restrictive abortion law. 

In Nigeria therefore, induced abortion is not carried out in public health institutions. Over 80% of induced abortion therefore end up in the hands of doctors in private setting and the rest are either self induced or by other health personnel and quacks.^14^

This study was therefore designed to define the characteristics of those admitted with induced abortion complications over a five year period.

## METHODS

This is a retrospective descriptive study carried out at University of Benin Teaching Hospital (UBTH). The study was approved by the hospital authority. The Medical Records of patients managed for induced abortion complications between Jan. 1^st^ 2009 and Dec. 31^st^ 2013 were reviewed. Relevant data relating to the socio-demographic profile of the patients, clinical presentation, abortion service providers and facilities and mode of termination of pregnancy, contraceptive awareness and uptake, type of immediate complications and management instituted and short term outcomes were manually extracted. Other gynaecological admissions and live births within the study period were equally extracted. All cases of complicated spontaneous abortions were carefully excluded from this study. EPI-INFO version 3.3 and FISHERS EXACT statistical packages were each used where appropriate to analyze the data. Test of significance was based on 95% confidence interval (p<0.05).

## RESULTS

During the study period, out of a total of 119 patients admitted and managed for induced abortion complications 111 case files were available for analysis giving a retrieval rate of 93.3%. During the period under review, 3547 patients were admitted into the gynaecological ward with complicated induced abortion constituting 3.4% of gynaecological admission.

**Fig.1:**
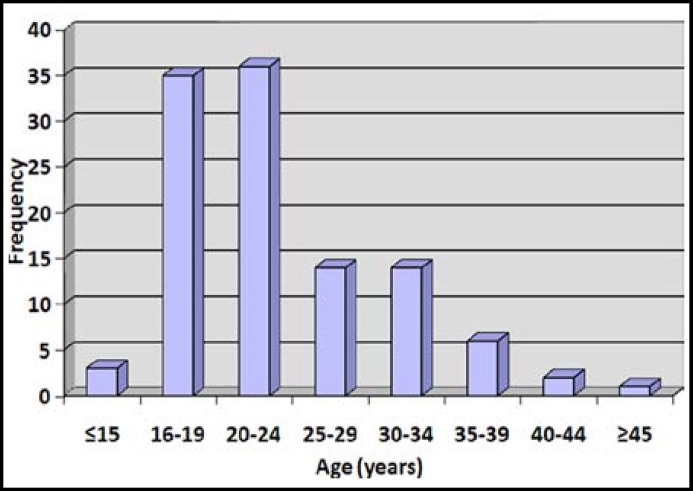
Simple bar chart showing age distribution of cases of complicated induced abortions.

At the same period there were 8391 live births in the centre giving a complicated induced abortion ratio of 14.2 per 1000 live births (Table-I). The ages of the patients ranged between 14 to 45 years with a mean of 23.5 ± 6.6 years while the modal age was 20-24year (32.8%) Fig.1.

The majority of the patients were nulliparous 78(70.3%) and 87 (78.4%) were single. Seventy one (64.0%) of the patients had at least one previous induced abortion. Most of the patients had secondary education 69(62.2%) while 27(24.5%) and 17(21.3%) had tertiary and primary education respectively. Over half of them were students 57(51.4%).

Among the abortion service providers medical doctors and chemists accounted for 64(57.7) and 19(17.1%) respectively. Other abortion service providers included Traditional Birth Attendants herbalists 3.6%, Nurse/midwives 9.9% and self 11.7%. Dilatation and curettage was the most used methods 45(40.5%). Other methods were manual vacuum aspiration 11(9.9%) oral medication10 (9.0%), vaginal pessaaries 10(9.0%) dilatation and evacuation 24 (21.6%), rupture of fetal membranes and Oxytocics 5 (4.5%) and rupture of fetal membranes only 4 (3.6%).

The gestational age at procurement of abortion ranged between 7 weeks and 27 weeks with a mean gestational age 12.8 ± 4.1 weeks and modal gestational age of 10 weeks 31(29.9%). Seventy (63.1%) of the procedure were carried out in the first trimester and the remaining 41(36.9%) were done in the second trimester.

The leading symptoms at presentation were lower abdominal pains 98(88.3%), vaginal bleeding 84 (75.7%), fever 63(56.8%) and offensive vaginal discharge 34(30.6%) The definitive diagnoses were made after clinical evaluation, laboratory and imaging investigations or at surgery. Retained products of conception 81 (73.0%) and postabortal sepsis 79 (71.2%) were the leading diagnoses Table-II.

All the patients received antibiotic coverage and evacuation of retained product of conception was done for 80(72.1%) of the patients (Table-III) the incidence of anaemia was 65.8% though only 50 (45.0%) of them received blood transfusion. Twenty (18.0%) of them had exploratory laparotomy with various procedures like drainage of pelvic abscess 13(11.7%), repair of perforated uterus 14(12.6%) and hysterectomy 5(4.5%). Ten (9.0%) patients had at least a course of renal dialysis. Duration of hospital stay ranged between 1 to 99 days, a mean of 9 ± 14.6 days with 65(58.6%) majority staying less than one week on admission.

Most 83(74.8%) of the patients were aware of contraception but only 27 (24.3%) had used any method. Eighty four (75.7%) of the patients improved and were discharged while 20(18.0%) mortality was recorded with a case fatality of 18%.Six of the deaths occurred within 24 hours of their admission another 5 in the succeeding 24 hours and the rest died thereafter. The remaining were either referred to other departments for further management 2(1.8%), discharged against medical advices 2(1.8%) or absconded 3(2.7%).

## DISCUSSION

In the reviewed period complicated induced abortion incidence of 14.2 per 1000 live births and 3.4% of gynaecological admission is lower than a similar report from this centre over 3 decades ago^15^ and it is significantly lower than the estimated rate of 100-140 per 1000 live births for the community.^5^ An increasing number of specialist clinics and private hospitals with improved services are the likely reasons for the disparity. This is attributable to the fact that only the complicated cases are seen in public health facilities in Nigeria with restrictive abortion laws. Again there are currently many alternative specialist facilities offering post abortion care in Benin City. It is obvious from this study that preventive efforts are not accessible to the most vulnerable groups. Efforts at reducing the rate of unplanned pregnancy by improving on the contraceptive uptake need not be over emphasized. The abortion service providers are mostly medical doctors in private settings. The contribution of the medical personnel to abortion complications has been previously documented.^16^^,^^17^

**Table-I:** Distribution of complicated induced abortion by years.

	**2003**	**2004**	**2005**	**2006**	**2007**	**Total**
Number of complicated induced abortions	23	13	11	18	46	111
Number of admission into gynaecological ward	757	716	471	785	818	3547
Number of live births	1543	1479	1512	1756	2101	8391
Percentage of gynaecological admission	3.0	1.8	2.3	2.3	5.6	3.1%
Ratio of complicated induced abortion per 1000 live births	14.9	8.8	7.3	10.3	21.9	13.2/1000

**Table-II:** Definitive diagnosis of patients admitted for complicated induced abortion

**Diagnosis**	**Number**	**Percentage**
Retained products of conception	81	73.0
Postabortal sepsis	79	71.2
Acute renal failure	14	12.6
Uremic encephalopathy	4	3.6
Abdomino pelvic abscess	12	10.8
Visceral perforation	11	9.9
Lower genital laceration	3	2.7
Hepatorenal syndrome	2	1.8
Disseminated intravascular coagulopathy (DIC)	2	1.8

**Table-III:** Distribution of treatment given to patients admitted for complicated induced abortion.

**Treatment**	**Number**	**Percentage**
Medical therapy only	21	18.9
Evacuation of retained products	79	71.2
Blood transfusion	50	45.0
Exploratory laparotomy	19	17.1
Examination under anaesthesia and repair of genital tract laceration	2	1.8
Renal dialysis	10	9.0

More than a half of the abortions took place in the first trimester which explains the over sixty percent of the procedures done by dilatation and curettage similar to previous reports^12^^, ^^15^. Only very few of the procedures were done by manual vacuum aspiration which has a leading safety profile. This calls for training and retraining of health care providers on this method to popularize its use.

Over 60% of these patients have had at least one previous termination of unwanted pregnancy and only 24.3% had used any form of contraception. This attests to high incidence of unplanned pregnancy and induced abortion in this environment^18^ consistent with high sexual activity with poor contraceptive use.^17^ It is a good clinical practice and expected that those seeking abortion care services should receive counselling on contraception from their service providers to improve on the contraceptive uptake and forestall the recurrence of unplanned pregnancies. This seems not to be true in this environment with the high incidence of previous abortion and disappointing low contraceptive use in this population.

 The prominent definitive diagnoses of retained products of conception and post abortion sepsis in this study attest to the level of unsafe abortion practices here in consonant with previous reports^16^. Those with post abortion sepsis and no retained products had antibiotic therapy as the main treatment. A significant number of the patients had exploratory laparotomy with evacuation of pelvic abscess, repair of uterine or bowel perforation or hysterectomy. This explains the burden of morbidity associated with this disorder.

Though the size of sample in this work can be considered a limiting factor, the immense contribution to maternal morbidity and mortality burdens by complicated induced abortion especially in developing countries is clearly demonstrated in this study with high maternal mortality ratio and case fatality. This is not different from other reports^2^. Late presentation, sepsis, abdominal visceral injury and renal failure were the leading contributory factors to this. The consequences of unsafe abortion as revealed in this work are a sad reminder of the poor state of the reproductive health system in developing countries. With the global increasing sexual activities coupled with unmet contraceptive need and restrictive abortion laws in the developing economies Nigeria inclusive, unsafe abortion will continue to impact adversely on womanhood.

There is a pressing need for an organised program for sexual and reproductive health education especially for the adolescents and unmarried sexually active segment of the society. This can be in-cooperated into the secondary and tertiary education curricula. Promotion of contraception to increase its uptake with particular emphasis on ‘Double Dutch’ option for the adolescents need not be overemphasized.

Training and continuing medical education for doctors who constitute the major service providers especially those favourably disposed to abortion services is highly indicated from this study. Provision of adequate and accessible health facilities for effective post abortion care services is a top priority here to check this scourge.

## Author‘s Contribution:

**Dr. M.E. Ikeanyi: **Conceived, designed and did statistical analysis

**Dr. C.A. Okonkwo: **Did review and final approval of manuscript, takes responsibility for all aspects of the work.

## References

[1] ( 1999). Reduction of maternal mortality; A Joint WHO/UNFPA UNICEF/World Bank Status.

[2] Unuigbe JA, Oronsaye AU, Orhue AAE (1988). Abortion related morbidity and mortality in Benin City, Nigeria, 1973 – 1985. Int J Gynaecol Obstet.

[3] Aboyeji AP Trends in maternal mortality in Ilorin, Nigeria 1987 - 1996. Int J Gynaecol Obstet.

[4] Okonofua FE (1997). Preventing unsafe abortion in Nigeria. Afr J Reprod Health.

[5] Whitaker C ( 1999). Safe abortion in Africa: ending the silence and starting a movement. Afr J Reprod Health.

[6] Okonofua FE, Friday Okonofua, Kunle Odunsi (2005). Abortion. Contemporary Obstetrics and Gynaecology for Developing Countries.

[7] World Health Organization (1992). The Prevention and Management of Unsafe AbortionReport of a Technical Working Group. WHO/MSM/92.5.

[8] World Health Organisation (WHO) (1993). Division of Family Health Maternal Health Management Health and Safe Motherhood Programme. The Prevention and Management of Unsafe Abortion. Report of a Technical Working Group Geneva 12 – 15 1992.

[9] World Health Organization (WHO) (2002). Technical and Policy. Guidance for Health System.

[10] Sedgh G, Henshaw S, Siagh S, Ahman E, Shah I H (2007). Induced Abortion; Estimated Rates and Trends Worldwide. Lancet.

[11] Strauss LT, Gamble SB, Parker WY, Cook DA, Zane SB (2006). Abortion Surveillance in United States, 2003. Surveillance Summaries.

[12] Henshaw SK, Singh S, Oye-Adeniran B (1998). Adewole IF. The incidence of induced abortion in Nigeria. Int Fam Plan Persped.

[13] Sedgh G, Bankola A, Oye – Adeniran B, Adewole I F, Singh S, Hussian R (2006). Unwanted pregnancy and associated features among Nigeria women. Int Fam Plan Persped.

[14] Okonofua FE, Odimegwu C, Ajabor H (1999). Assessing the prevalence and determinants of unwanted pregnancy in Nigeria.. Stud Fam Plan.

[15] Omu AE, Oronsaye AU, Faal MK (1981). Adolescent induced abortion in Benin City, Nigeria. Int J Gynaecol Obstet.

[16] Okonofua FE, Onwudiegwu U, Odunsi OA (1992). Illegal Induced abortion: A study of 74 cases in Ile Ife, Nigeria. Tropical Doctor.

[17] Adewole IF (1992). Trends in post abortion mortality and morbidity in Ibadan Nigeria. Int J Gynaecol Obstet.

[18] Oye-Adeniran B A, Adewole I F, Umoh, A V, Iwere N, Gbadeqesin A (2005). Induced abortion in Nigeria; Findings from focus discussion. Afr J Reprod Health.

